# Risk stratification for predicting postoperative recurrence/metastasis of colorectal cancer by grade of venous invasion coupled with histological subtype

**DOI:** 10.1186/s12876-022-02163-7

**Published:** 2022-02-23

**Authors:** Yasuo Imai, Masanori Ichinose

**Affiliations:** 1 Department of Diagnostic Pathology, Ota Memorial Hospital, SUBARU Health Insurance Society, 455-1 Oshima, Ota, Gunma 373-8585 Japan; 2 Department of Surgery, Shioya Hospital, International University of Health and Welfare, Tochigi, Japan

**Keywords:** Colorectal cancer, Histological subtype, Venous invasion, EVG staining, Recurrence, Metastasis, Stage II, Stage III, Adjuvant chemotherapy

## Abstract

**Background:**

Colorectal cancer (CRC) consists of several histological subtypes that greatly affect prognosis. Venous invasion (VI) has been implicated in the postoperative recurrence of CRC, but the relationship between the VI grade and postoperative recurrence in each histological subtype has not been clarified thus far.

**Methods:**

A total of 323 CRCs without distant metastasis at surgery (pathologic stage III or lower), including 152 well-to-moderately differentiated adenocarcinomas (WMDAs), 98 poorly differentiated adenocarcinomas (PDAs), and 64 mucinous adenocarcinomas (MUAs), were analyzed. They were routinely processed pathologically, and VI was graded as follows irrespective of location by elastica van Gieson staining: v0 (none), no venous invasion; v1 (mild), 1–3 invasions per glass slide; v2 (moderate), 4–6 invasions per glass slide; and v3 (severe), ≥ 7 invasions per glass slide. Filling-type invasion in veins with a minor axis of ≥ 1 mm increased the grade by 1. The association of VI grade with prognosis was statistically analyzed.

**Results:**

All recurrences occurred as distant metastases. Recurrence increased with VI grade in WMDA (v0 11.8%, v1 15.8%, v2 73.9%, v3 75.0%) and MUA (v0 15.2%, v1 30.8%, v2 40.0%). The recurrence rate was relatively high in PDA even with v0 and increased with VI grade (v0 27.8%, v1 32.7%, v2 33.3%, v3 60.0%). VI grade was a significant predictor of recurrence in WMDA but not in PDA and MUA by multivariate analysis. In node-negative (stage II or lower) CRC, the recurrence-free survival (RFS) rate exceeded 90% in v0 and v1 WMDA until postoperative day (POD) 2100 and v0 MUA until POD 1600 but fell below 80% in the other settings by POD 1000. In node-positive (stage III) CRC, the RFS rate fell below 80% in all histological subtypes by POD 1000.

**Conclusions:**

VI grade v1 had a similar recurrence rate and RFS as grade v0 and may not warrant adjuvant chemotherapy in node-negative (stage II or lower) WMDA. In addition to node-positive (stage III) CRC, adjuvant chemotherapy may be indicated for node-negative (stage II or lower) CRC when it is WMDA with VI grade v2 or v3, MUA with VI, or PDA.

**Supplementary Information:**

The online version contains supplementary material available at 10.1186/s12876-022-02163-7.

## Background

Colorectal cancer (CRC) is the third most common cancer and the second leading cause of cancer-related death worldwide [1]. The 5-year relative survival rates for colon cancer are 91% for the localized stage, 72% for the regional stage, 14% for the distant stage, and 63% for all stages combined, while those for rectal cancer are 89% for the localized stage, 72% for the regional stage, 16% for the distant stage, and 67% for all stages combined [2]. The localized stage means that there is no sign that cancer has spread outside of the colon and rectum, the regional stage means that cancer has spread outside of the colon and rectum to nearby structures or lymph nodes, and the distant stage means that cancer has spread to distant parts of the body, such as the liver, lung, or nonregional lymph nodes. Thus, the prognosis of metastatic CRC is poor.

Pathologic investigation of surgically resected specimens aims to provide information on factors that determine the need for adjuvant therapy. Venous invasion (VI) has been reported to be associated with an increased incidence of recurrence, especially hematogenous metastasis, and decreased overall survival [3]. In the Union for International Cancer Control (UICC) tumor, node, metastasis (TNM) staging system, V1 and V2 are defined as microscopic VI and macroscopic VI, respectively [4], but VI is not implicated in the stage definition of the UICC and American Joint Committee on Cancer (AJCC) TNM staging system [4, 5]. Furthermore, VI grade has not been defined to predict recurrence after surgery. Meanwhile, the histological subclassification of CRC has a great impact on prognosis. We previously showed that poorly differentiated adenocarcinoma (PDA) of CRC had significantly worse recurrence-free survival (RFS) and overall survival (OS) than well-to-moderately differentiated adenocarcinoma (WMDA) [6, 7]. VI and lymphatic invasion were found to be more frequent in PDA than in WMDA [6, 8]. The purpose of this study was to determine the criteria of VI that are likely to predict recurrence and metastasis after surgery in histological subtypes of CRC without distant metastasis at the time of surgery (pathologic stages 0, I, II, and III). VI was investigated by using an elastica van Gieson (EVG) staining which is feasible anywhere at low cost.

## Methods

### Patients

Consecutive patients with CRCs surgically resected at Tokyo Kosei Nenkin Hospital (TKNH) between April 1998 and March 2000 and International University of Health and Welfare, Shioya Hospital (IUHWSH) between 2012 and 2017 were included. Furthermore, patients with PDA, mucinous adenocarcinoma (MUA), and signet-ring cell carcinoma (SRCC) selected from all patients with CRCs surgically resected at Dokkyo Medical University Koshigaya Hospital (DMUKH) between 1990 and 2011, TKNH between 1991 and 2010, Saiseikai Kawaguchi General Hospital (SKGH) between 2000 and 2011, and IUHWSH between 2000 and 2011 were included in the study. All tumors in this study were diagnosed as primary CRCs. Patients with intraepithelial carcinoma that does not invade the lamina propria, appendiceal cancer, distant metastasis before and at the time of surgery, multiple synchronous invasive CRCs, asynchronous invasive CRC, and invasive cancer originating from other organs before and after surgery were excluded. Patients for whom the postoperative prognosis was unclear were also excluded. Clinicopathologic information was obtained from paper charts or through the electronic chart systems of the respective institutions. In principle, patient follow-up was performed every one to two months at the outpatient clinic for 5 years after surgery or until patients were referred to other institutions for family reasons or deteriorated performance status. Patients with pathologic stage III CRC were, in principle, administered adjuvant chemotherapy and were followed up weekly for the first 6 months. However, older patients with poor performance status were administered milder chemotherapeutic regimens and were followed up monthly. Blood tests and abdominal ultrasonography were performed every 2 months, and contrast-enhanced computed tomography and colonoscopy were performed every 6 months for the first year and yearly for 4 more years. This study was conducted in accordance with the Declaration of Helsinki, and this study protocol was approved by the ethical review boards of the participating hospitals: DMUKH, Koshigaya 23008; TKNH, 30/11/2011; SKGH, no. 24-5: IUHWSH, FK-94.

### Histopathologic examination

**Fig. 1:**
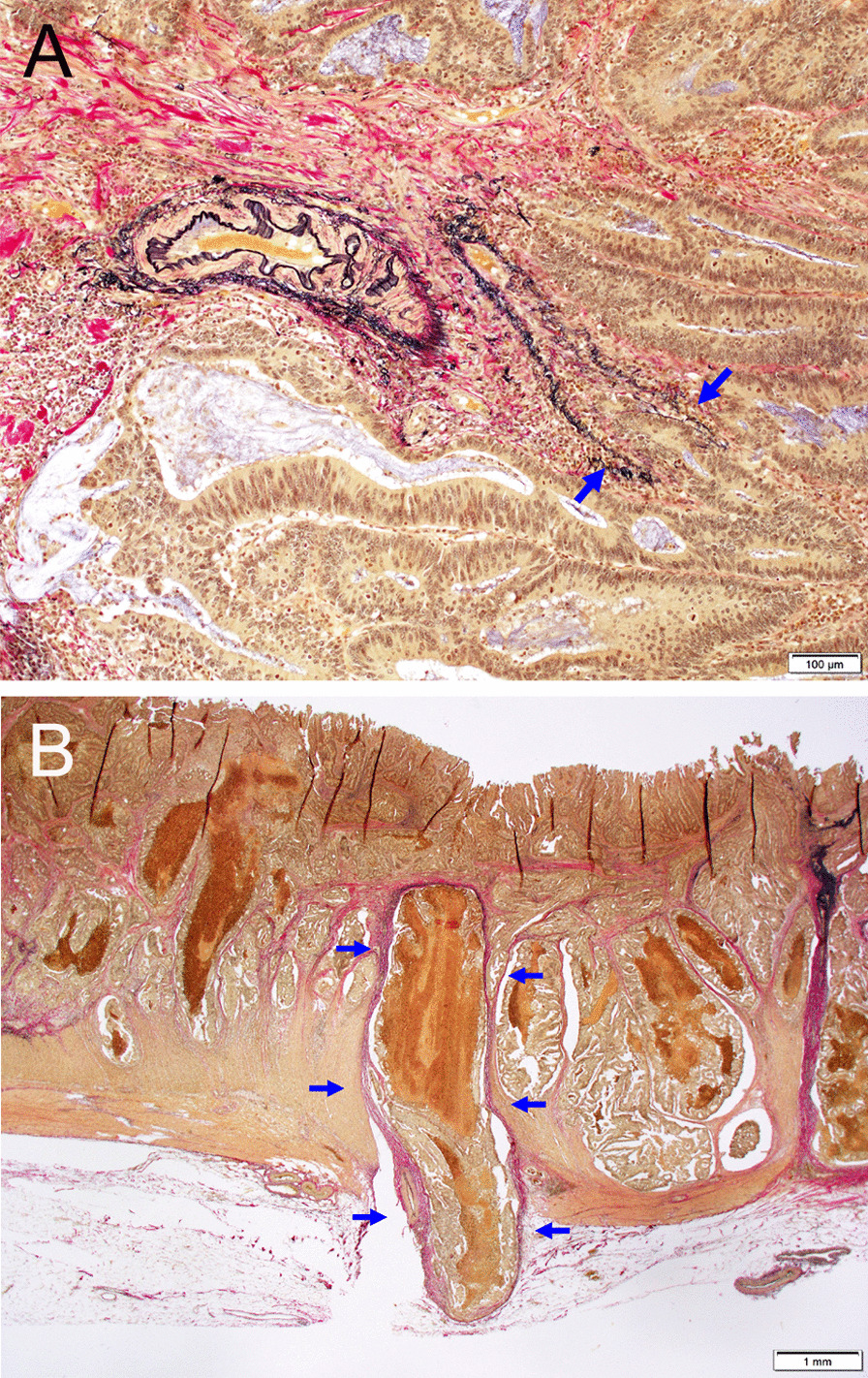
Representative histopathology of venous invasion. **A** Infiltrating type of venous invasion. The cancer cell nest destroys and invades the circular elastic plate adjacent to the artery. **B** Filling type of venous invasion. Tumor embolus is found in the vein with a diameter greater than 1 mm

All surgical specimens were routinely processed for pathologic diagnosis. Putative early cancer at gross diagnosis (putative pTis and pT1) was subjected to microscopic investigation of the whole tumor area. In putative advanced cancer (putative pT2–4), the maximal cut surface of the tumor, involving the transition between the tumor and normal mucosa, and the cut surface involving the deepest tumor penetration were microscopically investigated. Clinicopathologic classifications and stage groupings were performed based on the World Health Organization (WHO) classification of colorectal tumors (4th ed.) and the AJCC TNM staging system (7th ed.) [5, 9]. EVG staining was performed in two or more, if necessary, sections that included the deepest tumor penetration and the area of transition between the tumor and normal mucosa. Each section usually contained approximately 2 to 5 cm^2^ of tissue per glass slide. When tumor cells invaded or were located in the tubular structure formed by an elastic plate adjacent to the artery, which means an adventitia of the vein, VI was diagnosed (Fig. 1A). VI in each section was graded according to the number of VIs irrespective of the location of the veins: v0 (none), no venous invasion; v1 (mild invasion), 1 to 3 venous invasions per glass slide; v2 (moderate invasion), 4 to 6 venous invasions per glass slide; and v3 (severe invasion), ≥ 7 venous invasions per glass slide. When filling-type VI, in which tumor cells filled the vascular lumen, was found in a macroscopically identifiable vein with a minor axis of ≥ 1 mm (Fig. 1B), the grade of a v1 or v2 was raised by 1. The VI grade in each case was based on the maximal grade in the investigated sections.

### Statistical analysis

Specific parameters between two patient cohorts and associations between two variables were compared using Fisher’s exact test (2 × 2 contingency tables) or the chi-square test with or without Yates’ correction (other contingency tables). Univariate analysis was performed using Fisher’s exact test (2 × 2 contingency tables) or the chi-square test with or without Yates’ correction (other contingency tables) to investigate associations between parameters and recurrence. Multivariate logistic regression analysis was performed on parameters with *p* values < 0.10 in univariate analysis. Survival curves were depicted using the Kaplan–Meier method, and differences between survival curves were analyzed by the log-rank test. A *p* value < 0.05 was considered significant. Statistical analyses were performed using IBM SPSS Statistics 20 (IBM Corp, Armonk, NY, USA).

## Results

### Clinicopathologic features of each histological subtype

A total of 323 CRCs, comprising 152 WMDAs, 98 PDAs, 64 MUAs, and nine SRCCs, were included in this study. The follow-up periods from surgery to cancer-related death or censoring of WMDA, PDA, MUA, and SRCC patients were 17–4936 days (median: 1842.5 days), 15–4026 days (median: 667 days), 0–4860 days (median: 1341 days), and 96–3243 days (median: 1648 days), respectively. Female was significantly more frequent than male in PDA (52.0%) as compared with WMDA (37.5%) (*p* = 0.027) and MUA (34.4%) (*p* = 0.036), respectively. Right-sided location was significantly more frequent than left-side location in PDA (59.2%) and MUA (48.4%) compared with WMDA (29.6%) (*p* < 0.001 and *p* = 0.012, respectively). The depth of invasion was significantly greater in PDA and MUA than in WMDA (pTis/pT1/pT2 vs. pT3/pT4: *p* < 0.001 and *p* = 0.006, respectively). VI (v1-3) and nodal metastasis (pN1/pN2) were significantly more frequent in PDA than in WMDA (*p* < 0.001 and *p* < 0.001, respectively). Pathologic TNM stage was significantly higher in PDA than in WMDA (stage 0/I/II vs. stage III: *p* < 0.001). The results of clinicopathologic analyses are summarized in Table 1.

**Table 1:** Patients' clinicopathologic features at surgery

	WMDA (n = 152)	PDA (n = 98)	MUA (n = 64)	SRCC (n = 9)
Age				
Range	34–93	36–92	26–90	30–82
Median	69	69.5	71	71
Sex				
Male	95	47	42	5
Female	57	51	22	4
Family history of CRC				
Yes	7	1	4	1
No	145	96	59	8
Unknown		1	1	
Location				
Left-sided	107	40	33	4
Right-sided	45	58	31	5
Depth of invasion				
pTis/pT1/pT2	3/15/22	0/2/3	0/1/5	0/0/1
pT3/pT4	92/20	57/36	45/13	2/6
Venous invasion				
Yes (v1-3)	84	80	31	8
No (v0)	68	18	33	1
Lymphatic invasion				
Yes	55	86	38	8
No	88	12	26	1
Unknown	9			
Nodal metastasis				
pN1/pN2	41/17	37/30	17/9	2/4
pN0	94	31	38	3
pTNM stage				
0/I/II	3/33/58	0/4/27	0/4/33	0/1/1
III	58	67	27	7
Postoperative chemotherapy				
Yes	52	48	22	3
No	100	48	39	6
Unknown		2	3	
Postoperative radiation				
Yes	5	2	3	1
No	147	94	58	8
Unknown		2	3	

*WMDA* well-to-moderately differentiated adenocarcinoma, *PDA* poorly differentiated adenocarcinoma, *MUA* mucinous adenocarcinoma, *SRCC* signet-ring cell carcinoma, *pTNM* pathologic tumor, node, metastasis

### Postoperative recurrence and prognosis in each histological subtype

**Fig. 2:**
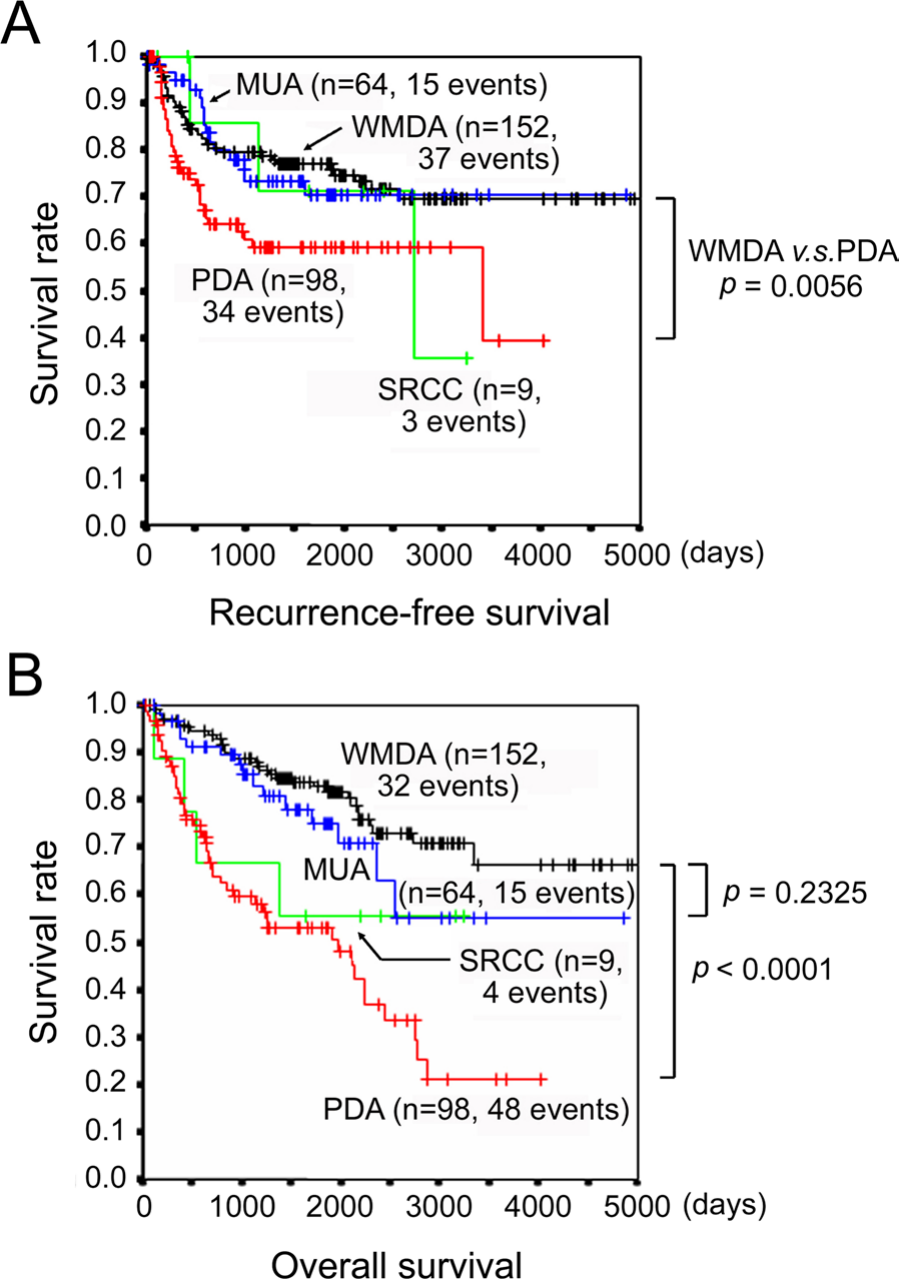
Prognoses of CRC histological subtypes. **A** Recurrence-free survival. **B** Overall survival. CRC, colorectal cancer; WMDA, well-to-moderately differentiated adenocarcinoma; PDA, poorly differentiated adenocarcinoma; MUA, mucinous adenocarcinoma; SRCC, signet-ring cell carcinoma

In this study cohort, local recurrence was not found in any case throughout the histological subtypes. Therefore, recurrence means distant metastasis due to hematogenous and/or lymphatic spread or peritoneal dissemination. Recurrence was found in 37 of 152 (24.3%) WMDA patients, 34 of 98 (34.7%) PDA patients, 15 of 64 (23.4%) MUA patients, and 3 of 9 (33.3%) SRCC patients. The recurrence rate was marginally higher in PDA than in WMDA (relative risk (RR) 1.425, 95% confidence interval (CI): 0.963–2.094) (Table 2 and Additional file 1: Table S1). Meanwhile, the recurrence rate was similar between WMDA and MUA (RR 0.963, 95% CI: 0.563–1.592). Survival curve analyses revealed that RFS and OS were both significantly worse in PDA than in WMDA (Fig. 2). WMDA and MUA revealed similar RFS, but OS was somewhat worse in MUA than in WMDA, although not statistically significant.

**Table 2:** Recurrence rate according to the grade of venous invasion

	Grade of venous invasion				Total
	v0	v1	v2	v3	
WMDA					
*Recurrence (%)	8/68 (11.8%)	9/57 (15.8%)	17/23 (73.9%)	3/4 (75.0%)	37/152 (24.3%)
RR (95% CI)	Reference	1.342 (0.565–3.200)	6.283 (3.364–10.834)	6.375 (2.209–9.023)	
**p value		0.604	< 0.001	0.010	
Median DTR (range)	438.5 (268–1264)	593 (105–2212)	232 (18–2561)	300 (51–368)	374 (18–2561)
PDA					
Recurrence (%)	5/18 (27.8%)	17/52 (32.7%)	6/18 (33.3%)	6/10 (60.0%)	34/98 (34.7%)
RR (95% CI)	Reference	1.177 (0.555–2.839)	1.200 (0.455–3.217)	2.160 (0.874–4.695)	
*p* value		0.776	1.000	0.125	
Median DTR (range)	608 (195–3407)	341 (123–1070)	207.5 (139–539)	142.5 (87–297)	244 (87–3407)
MUA					
Recurrence (%)	5/33 (15.2%)	8/26 (30.8%)	2/5 (40.0%)		15/64 (23.4%)
RR (95% CI)	Reference	2.031 (0.781–5.423)	2.640 (0.645–7.310)		
*p* value		0.209	0.223		
Median DTR (range)	547 (423–1600)	582.5 (22–993)	633.5 (282–985)		572 (22–1600)
SRCC					
Recurrence (%)	0/1 (0.0%)	2/6 (33.3%)	0/1 (0.0%)	1/1 (100.0%)	3/9 (33.3%)
RR (95% CI)	Reference	N.A	N.A	N.A	
*p* value					
Median DTR (range)		785.5 (436–1135)		2710 (2710–2710)	1135 (436–2710)

*WMDA* well-to-moderately differentiated adenocarcinoma, *PDA* poorly differentiated adenocarcinoma, *MUA* mucinous adenocarcinoma, *SRCC* signet-ring cell carcinoma, *RR* relative risk, *CI* confidence interval, *DTR* days to recurrence, *N.A* not applicable, *v0* no venous invasion, *v1* 1–3 invasions/glass slide, *v2* 4–6 invasions/glass slide, *v3* ≥ 7 invasions/glass slide

The filling type of venous invasion in a macroscopically identifiable vein with a minor axis of ≥ 1 mm raised the grade of a v1 or v2 by 1

*In this study cohort, there were no cases of local recurrence throughout the histological subtypes. Therefore, recurrence means distant metastasis due to hematogenous/lymphatic spread or peritoneal dissemination

**Statistical analysis was performed by Fisher's exact test

### Recurrence rate according to the VI grade in each histological subtype

The recurrence rate of WMDA was similar between v0 (11.8%) and v1 (15.8%), but it was significantly higher in v2 (73.9%) and v3 (75.0%) than in v0. The recurrence rate of PDA was similar among v0 (27.8%), v1 (32.7%) and v2 (33.3%), but it was marginally higher in v3 (60.0%) than in v0. The recurrence rate of MUA with v1 (30.8%) and v2 (40.0%) was approximately twice as high as that with v0 (15.2%), but the difference did not reach a statistical significance. The recurrence rates of WMDA with v0 and v1 and MUA with v0 were in the 10% range, but those in other settings of WMDA, MUA and PDA were approximately 30% or more. These results are summarized in Table 2.

### Prognostic factors for recurrence in each histological subtype

Associations between recurrence and clinicopathologic parameters were investigated by Fisher’s exact test or the chi-square test as a univariate analysis of recurrence-predictive factors. Depth of tumor invasion, VI grade, nodal metastasis, and pathologic TNM stage were candidate predictors of WMDA, and depth of tumor invasion, nodal metastasis, and pathologic TNM stage were candidate predictors for PDA. Postoperative chemotherapy was found to be significantly associated with recurrence in WMDA, PDA, and MUA and postoperative radiotherapy was found to be significantly associated with recurrence in WMDA. However, they were not deemed candidate recurrence-predictive factors because they were performed as salvage therapy after recurrence. There were no candidate predictors for MUA and SRCC. These results are summarized in Table 3.

**Table 3:** Associations between recurrence and clinicopathologic parameters

Histotype	Parameter	Odds ratio	95% CI	Statistics	*p* value
WMDA (n = 152)	Age (≥ 65)	1.310	0.587–2.919	F	0.556
	Sex (male)	0.542	0.256–1.148	F	0.121
	Location (right)	1.405	0.639–3.091	F	0.413
	Depth of invasion	N.C	N.C	C	0.000
	Venous invasion	N.C	N.C	C	0.000
	Nodal metastasis (positive)	6.131	2.715–13.842	F	0.000
	pTNM Stage	N.C	N.C	C	0.000
	Postoperative chemtherapy (done)	18.117	7.043–46.604	F	0.000
	Postoperative irradiation (done)	N.C	N.C	F	0.001
PDA (n = 98)	Age (≥ 65)	0.846	0.357–2.005	F	0.825
	Sex (male)	0.658	0.284–1.524	F	0.397
	Location (right)	1.179	0.503–2.761	F	0.830
	Depth of invasion	N.C	N.C	C	0.058
	Venous invasion	N.C	N.C	C	0.346
	Nodal metastasis (positive)	2.991	1.084–8.252	F	0.040
	pTNM Stage	N.C	N.C	C	0.065
	Postoperative chemtherapy (done)	3.800	1.549–9.322	F	0.005
	Postoperative irradiation (done)	N.C	N.C	F	0.123
MUA (n = 64)	Age (≥ 65)	0.882	0.257–3.029	F	1.000
	Sex (male)	1.063	0.312–3.614	F	1.000
	Location (right)	1.841	0.568–5.970	F	0.382
	Depth of invasion	N.C	N.C	C	0.320
	Venous invasion	N.C	N.C	C	0.246
	Nodal metastasis (positive)	2.824	0.860–9.269	F	0.132
	pTNM Stage	N.C	N.C	C	0.201
	Postoperative chemtherapy (done)	8.750	2.314–33.080	F	0.001
	Postoperative irradiation (done)	6.923	0.581–82.548	F	0.147
SRCC (n = 9)	Age (≥ 65)	0.100	0.004–2.504	F	0.226
	Sex (male)	0.250	0.013–4.729	F	0.524
	Location (right)	2.000	0.112–35.807	F	1.000
	Depth of invasion	N.C	N.C	C	1.000
	Venous invasion	N.C	N.C	C	0.392
	Nodal metastasis (positive)	N.C	N.C	F	0.464
	pTNM Stage	N.C	N.C	C	0.526
	Postoperative chemtherapy (done)	10.000	0.399–250.419	F	0.183
	Postoperative irradiation (done)	N.C	N.C	F	1.000

In this study cohort, there were no cases of local recurrence throughout the histological subtypes. Therefore, recurrence means distant metastasis due to hematogenous/lymphatic metastasis or peritoneal dissemination

*WMDA* well-to-moderately differentiated adenocarcinoma, *PDA* poorly differentiated adenocarcinoma, *MUA* mucinous adenocarcinoma, *SRCC* signet-ring cell carcinoma, *pTNM* pathologic tumor, node, metastasis, *CI* confidence interval, *N.C* not calculated, *F* Fisher's exact test, *C* chi-square test

Then, multivariate analysis was performed by using logistic regression analysis in WMDA and PDA. Pathologic TNM stage was excluded from the analysis because it was determined based on the depth of tumor invasion and nodal metastasis. VI grade, depth of tumor invasion, and nodal metastasis were all significant predictive factors of recurrence in WMDA. Depth of tumor invasion was the only significant predictive factor in PDA. These results are summarized in Table 4.

**Table 4:** Multivariate logistic regression analysis for predicting recurrence

Histotype	Parameter	Odds ratio	95% CI	*p* value
WMDA	Venous invasion	2.879	1.578–5.252	0.001
	Depth of invasion	2.881	1.281–6.479	0.011
	Nodal metastasis (positive)	3.113	1.249–7.759	0.015
PDA	Depth of invasion	2.542	1.110–5.822	0.027
	Nodal metastasis (positive)	2.213	0.767–6.381	0.142

In this study cohort, there were no cases of local recurrence throughout the histological subtypes. Therefore, recurrence means distant metastasis due to hematogenous/lymphatic metastasis or peritoneal dissemination

*WMDA* well-to-moderately differentiated adenocarcinoma, *PDA* poorly differentiated adenocarcinoma, *CI* confidence interval

### Recurrence in CRC histological subtypes with or without nodal metastasis

**Fig. 3:**
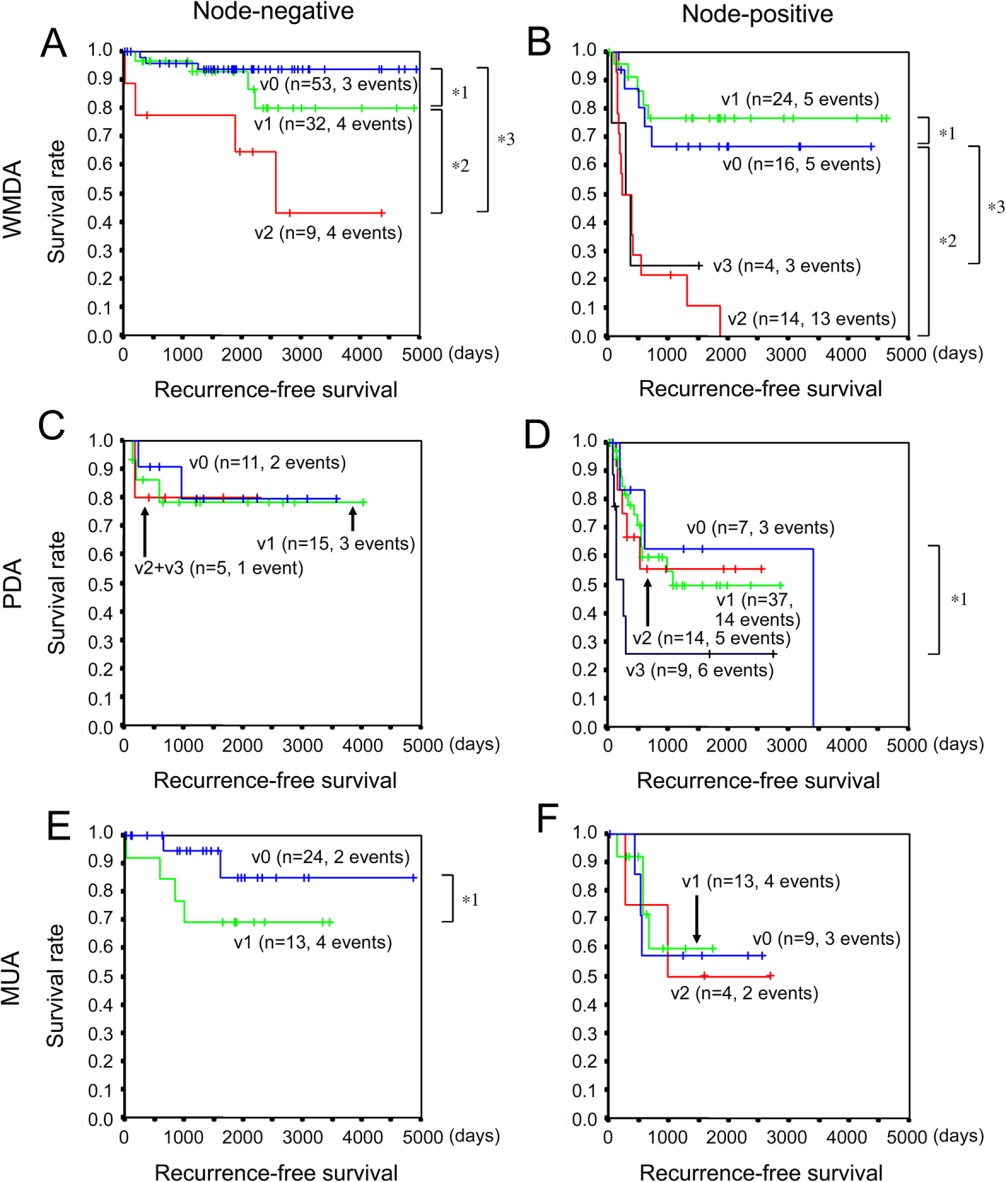
Recurrence-free survival of CRC histological subtypes with or without nodal metastasis. **A** WMDA without nodal metastasis. *1, *p* = 0.2676. *2, *p* = 0.0603. *3, *p* = 0.0011. **B** WMDA with nodal metastasis. *1, *p* = 0.5539. *2, *p* = 0.0002. *3, *p* = 0.0461. **C** PDA without nodal metastasis. **D** PDA with nodal metastasis. *1, *p* = 0.0944. **E** MUA without nodal metastasis. *1, *p* = 0.1635. **F** MUA with nodal metastasis. CRC, colorectal cancer; WMDA, well-to-moderately differentiated adenocarcinoma; PDA, poorly differentiated adenocarcinoma; MUA, mucinous adenocarcinoma

In this section, the effect of VI grade on recurrence was investigated in CRC histological subtypes with or without nodal metastasis by the Kaplan–Meier method and the long-rank test. In this study, node-negative CRC and node-positive CRC correspond to stage II or lower and stage III of the TNM classification, respectively. In the analysis of node-negative WMDA, there was no significant difference in clinicopathologic parameters between v0 and v1 or v2 except for male predominance in v2 cases (Additional file 1: Table S2). Comparison of Kaplan–Meier curves revealed that RFS was significantly poorer in v2 cases than in v0 cases, while v0 and v1 had similar prognoses (Fig. 3A). The recurrence rates of v0 and v1 were quite low (5.7% and 12.5%, respectively: *p* = 0.417 by Fisher’s exact test), and the RFS rates of v0 and v1 exceeded 90% until postoperative day (POD) 2100. In the analysis of node-positive WMDA, there was no significant difference in clinicopathologic parameters between v0 and v1, v2, or v3 (Additional file 1: Table S3). The Kaplan–Meier curve analysis revealed that RFS was similar between v0 and v1, but the RFS of v2 or v3 was significantly poorer than that of v0 (Fig. 3B). The RFS rate was less than 80% by POD 1000 in all VI grades. The prognosis of v0 of node-positive WMDA was poorer than that of v0 or v1 of node-negative WMDA (Fig. 3A, B).

In the analysis of node-negative PDA, there was no significant difference in clinicopathologic parameters between v0 and v1 or v2 + v3 except for younger age and left-sided location in v2 + v3 cases (Additional file 1: Table S4). By Kaplan–Meier curve analysis, v0, v1, and v2 + v3 had similar RFS rates, which fell below 80% by POD 1000 (Fig. 3C). In the analysis of node-positive PDA, there was no significant difference in clinicopathologic parameters between v0 and v1, v2, or v3 (Additional file 1: Table S5). The Kaplan–Meier curve analysis revealed that RFS was similar among v0, v1, and v2 but that of v3 was poorer (Fig. 3D). The RFS rate fell below 80% in all VI grades by POD 1000.

In the analysis of node-negative MUA, there was no significant difference in clinicopathologic parameters between v0 and v1 (Additional file 1: Table S6). The RFS rate exceeded 90% in v0 until POD 1600, and the RFS of v1 was marginally poorer than that of v0 (Fig. 3E). In the analysis of node-positive MUA, there was no significant difference in clinicopathologic parameters between v0 and v1 or v2 (Additional file 1: Table S7). The Kaplan–Meier curve analysis revealed that v0, v1, and v2 had similar RFS rates, which fell below 80% by POD 1000 (Fig. 3F).

Survival curve analysis was not performed in SRCC because of the small number of cases.

## Discussion

In the present study, we found that the frequency of VI and its effect on postoperative recurrence/metastasis differed depending on the histological subtype. Possible applications of our results to postoperative treatment are discussed below.

In routine surgical pathology, histological subtype, grade of differentiation, depth of tumor invasion, lymphovascular invasion, lymph mode metastasis, and resection margin status are evaluated in the resected CRC specimens. Clinicians plan postoperative treatment based on the pathology report. Metastasis is caused by tumor cell spread via lymphatics or veins or by dissemination. VI is theoretically a risk factor for distant metastasis, but it is not a defining factor for TNM stage [4, 5]. Meanwhile, some investigators reported that VI was a significant prognostic factor independent of depth of invasion, grade, and lymph node metastasis [10–12]. The prediction of recurrence based on the VI degree by the EVG staining is inexpensive and does not require special equipment in contrast to the molecular testing. It is an inspection that is feasible at any facility worldwide. Since the incidence of VI increases with increasing grade [13], we investigated the clinical impact of VI for each histological subtype and discussed the indications for adjuvant therapy depending on the VI grade.

In Japan, VI has long been classified as v0 (none), v1 (mild), v2 (moderate), and v3 (severe) based on the pathologist’s subjectivity until the former Japanese Classification of Colorectal Carcinoma (8th edition) [14]. The latest Japanese Classification of Colorectal Carcinoma (9th edition) and its English version (3rd edition) classified VI as V0 (none), V1a (microscopic VI, mild), V1b (microscopic VI, moderate), and V1c (microscopic VI, severe) based on the pathologist’s subjectivity and V2 (macroscopic VI) [15, 16]. This revision may reflect the UICC TNM staging system classification (8th edition), in which VI is classified either in V1 (microscopic VI) or V2 (macroscopic VI) [4]. Metastasis is more likely to occur when the primary tumor growth spreads into large veins than when only small venules are invaded [17, 18]. However, some authors reported that the differences between VI involving a large invaded vein and VI involving a small invaded vein were not significant [3]. VI was also subdivided by the number of invaded veins. The number of VIs was subdivided into 0 (G0), 1–3 (G1), and 4 or more (G2) per glass slide by Sato et al. [3]; 0 (V0), 1–3 (V1), 4–6 (V2), and 7 or more (V3) per glass slide by Shirouzu et al. [19]; 0, 1–2 (minimal), 3–4 (intermediate), and 5 or more (massive) per glass slide by Sternberg et al. [20]; 0 (v0), 1–2 (v1), 3–5 (v2), and 6 or more (v3) per glass slide by Ouchi et al. [21]; and 0 (v0), 1–2 (v1), 3–6 (v2), and 7 or more (v3) per glass slide by Yokokawa et al. [22]. The number of VIs was found to impact on overall survival [3, 19, 21], local recurrence [19], and liver metastasis [19, 22]. What was of interest was that G0 and G1 by Sato et al. had similar OS rates in stage II CRC [3] and that V0 and V1 by Shirouzu et al. had similar OS rates in CRC with Dukes’ stage B and C [19]. Meanwhile, Sternberg et al. reported that only minimal VI is required for the seeding of clinically relevant hematogenous metastases [20].

In addition, VI is subclassified by the morphologic type and location of the involved veins. In previous studies, VI was classified into the filling type, the floating type in which tumor cells did not adhere to the vein and floated in the lumen, and the infiltrating type in which tumor cells were seen infiltrating the wall of the vessel [3, 19, 20, 23]. These types often coexist [19, 20], and the survival impact between the filling type and nonfilling type was not significant [3, 23]. With respect to the location of the venous vessels involved, there is a controversy over the prognostic impact of intramural and extramural VI. Some authors have concluded that extramural VI indicates poor prognosis [11, 17, 18, 24], but others have shown that both intramural and extramural VI can be a cause of poor prognosis [12, 20]. In addition, Sato et al. did not find a significant difference between intramural and extramural VI [3]. Since the intramural vein and extramural vein are connected, we considered that there may be little reason to distinguish them with respect to metastatic potential. In clinical practice, the latest Japanese Classification of Colorectal Carcinoma (9th edition) and its English version (3rd edition) recommend recording the level of the deepest vascular invasion, such as V1a (SS), in which SS means subserosa [15, 16].

Given the above studies and recommendations, we graded venous invasion based on the number of venous invasions and the size of the invaded vein. Microscopic VI and macroscopic VI were integrated to create a simple grading system, as with the former Japanese Classification of Colorectal Carcinoma (8th and older editions) [14]. The VI grade was increased by 1 when the filling type of macroscopic VI (Fig. 1B) was observed because we reasoned that such a situation might have greater metastatic potential than the infiltrating type of microscopic VI (Fig. 1A).

To the best of our knowledge, all similar studies analyzed CRC without histopathologic subtyping. However, CRC consists of various histological subtypes. For example, 257 consecutive CRCs resected between April 1998 and March 2000 in TKNH and between 2012 and 2017 in IUHWSH comprised 228 WMDAs (88.7%), 17 PDAs (6.6%), 10 MUAs (3.9%), one SRCC (0.4%), and one neuroendocrine carcinoma (0.4%). Thus, histological subtypes other than WMDA comprise approximately 10% of CRC and exhibit distinct biological characteristics, leading to a more unfavorable prognosis than WMDA [6–8]. Their treatment strategies should not be the same as that for WMDA.

In the present study, we investigated CRCs without distant metastasis at the time of surgery, i.e., pathologic stage III or lower. The rate of postoperative recurrence was marginally higher in PDA than in WMDA, and the rate of recurrence was similar between WMDA and MUA (Additional file 1: Table S1). Kaplan–Meier curve analysis revealed that RFS was significantly worse in PDA than in WMDA and that RFS was similar between WMDA and MUA (Fig. 2). Due to the small number of cases, SRCC was not subject to a statistical analysis in this case. These data suggested that recurrence predictive factors for WMDA and PDA should be determined independently. In fact, the rate of recurrence depending on the VI grade was different for each histological subtype. Then, multivariate analyses for recurrence predictive factors revealed that VI grade was a significant prognostic factor in WMDA but not in PDA, MUA, or SRCC. We speculate that these histological subtypes themselves represent strong invasiveness and may have masked the effect of VI.

Adjuvant chemotherapy is the standard of care for stage III CRC after resection [25]. Recurrence occurred in approximately 30% of stage III CRCs resected between 1996 and 2001, and the estimated RFS at five years was 70% in the United States [26]. After surgical resection of stage III CRC, adjuvant chemotherapy provides a 22–32% OS advantage and a 30% RR reduction in disease recurrence [27]. Meanwhile, recurrence occurred in 10.0% of stage II CRC resected between 1995 and 2007, and the estimated RFS at five years was 90% [26]. The usefulness of adjuvant therapy for stage II disease remains controversial [25]. Currently, the American Society of Clinical Oncology (ASCO), the European Society of Medical Oncology (ESMO), and the US National Comprehensive Cancer Network (NCCN) have designated high-risk stage II CRCs as cases having any one or more of the following characteristics: stage pT4, poorly differentiated tumor, mucinous tumor, perforation, lymphovascular invasion, perineural invasion, a small number of lymph nodes examined (ASCO < 13; ESMO and NCCN < 12), and close, indeterminate, or positive margins after surgery (NCCN) [28]. However, no specific numerical criteria have been established for lymphovascular invasion. Thus, there is still no answer as to whether or not adjuvant therapy should be performed when mild venous invasion, such as one venous invasion of infiltrating type per glass slide, is observed in stage II CRC. Accordingly, we next analyzed the RFS of stage 0/I/II (node-negative) and stage III (node-positive) disease separately in each histological subtype. In node-negative CRCs, the RFS rate exceeded 90% in v0 and v1 WMDA for more than 5 years and v0 MUA for more than 4 years after surgery (Fig. 3A, E), but the RFS rate fell to around 80% in PDA irrespective of VI grade within 3 years after surgery. These results suggest that the RFS of WMDA with v0 and v1 and MUA with v0 are similar but the RFS in the other settings is more unfavorable. If a 5-year RFS rate of over 90% is deemed satisfactory, adjuvant chemotherapy could be spared for WMDA with v0 and v1 and MUA with v0. However, it may be better to consider adjuvant chemotherapy for WMDA with v2 and v3 as well as MUA with VI and all PDAs. On the other hand, in node-positive CRCs, the RFS rate fell below 80% in WMDA, PDA, and MUA irrespective of the VI grade within 3 years after surgery. These results suggest that adjuvant therapy is warranted for all histological subtypes of stage III CRC.

Our study has some limitations. First, this was a retrospective study. Second, the patient groups were rather small after stratification. Therefore, our results must be evaluated in a larger prospective study. Third, adjuvant chemotherapy was performed in a subset of included patients, approximately 20–30% of node-negative (stage II or lower) patients and 50–60% of node-positive (stage III) patients (Additional file 1: Tables S2 to S7). The recurrence rates without adjuvant chemotherapy would have been higher than those observed in this study. For example, 30% of patients with stage III CRC benefit from standard adjuvant chemotherapy, 50% of them are already cured by the surgery and 20% of them experience disease recurrence despite adjuvant treatment [29]. However, the rate of postoperative chemotherapy between v0 and v1, v2, or v3 did not differ significantly in any node-negative and node-positive histological subtypes (Additional file 1: Tables S2 to S7), and we speculate that postoperative chemotherapy would not significantly affect our conclusions. Fourth, the effect of neural invasion by cancer was not evaluated in this study. In Japan, a description of neural invasion was not common until July 2013, when the former Japanese Classification of Colorectal Carcinoma (8th edition) was published [14]. Although neural invasion may be a risk factor for recurrence, we infer that cancer extension along neural fibers would result in local recurrence. Meanwhile, all recurrences occurred as distant metastases in this study. In addition, neural invasion was noted only in three (3.1%) of 98 consecutive CRC cases resected at IUHWSH between 2015 and 2017. We therefore speculate that the effect of neural invasion on prognosis might be minimal in this study. Fifth, tumor budding, which is a small cluster of fewer than five cells at the invasive front of the tumor, was not analyzed in this study. Although it has been reported to be an independent adverse prognostic factor in stage II CRC [30], tumor budding has not been included in the AJCC TNM staging system thus far. Furthermore, assessment of tumor budding may not be applicable in some histological subtypes, such as MUA and SRCC [30].

## Conclusions

VI degree was a significant predictor of recurrence in WMDA, which comprises nearly 90% of consecutively resected CRCs, but not in other histological subtypes, by multivariate analysis. VI grade v1 might not warrant adjuvant chemotherapy in node-negative (stage II or lower) WMDA. In addition to node-positive (stage III) CRC, adjuvant chemotherapy may be indicated for node-negative CRC when it is WMDA with VI grade v2 or v3, MUA with VI, or PDA.

## Supplementary Information

**Additional file 1:Tables S1–S7.** Frequencies of metastasis and clinicopathologic characteristics in each histological subtype with or without nodal metastasis.

## Data Availability

All data generated or analyzed in this study are included in this published article and an additional file.

## Abbreviations

CRC: Colorectal cancer
VI: Venous invasion
UICC: Union for International Cancer Control
TNM: Tumor, node, metastasis
AJCC: American Joint Committee on Cancer
PDA: Poorly differentiated adenocarcinoma
RFS: Recurrence-free survival
OS: Overall survival
WMDA: Well-to-moderately differentiated adenocarcinoma
EVG: Elastica van Gieson
TKNH: Tokyo Kosei Nenkin Hospital
IUHWSH: International University of Health and Welfare, Shioya Hospital
MUA: Mucinous adenocarcinoma
SRCC: Signet-ring cell carcinoma
DMUKH: Dokkyo Medical University Koshigaya Hospital
SKGH: Saiseikai Kawaguchi General Hospital
WHO: World Health Organization
RR: Relative risk
CI: Confidence interval
POD: Postoperative day
ASCO: American Society of Clinical Oncology
ESMO: European Society of Medical Oncology
NCCN: National Comprehensive Cancer Network
