# Supplementary material for: Risk stratification for predicting postoperative recurrence/metastasis of colorectal cancer by grade of venous invasion coupled with histological subtype
Source: BMC Gastroenterol. 2022 Feb 23;22:79. doi: 10.1186/s12876-022-02163-7 (PMC8867649; doi:10.1186/s12876-022-02163-7)
Supplement: Supplementary file 1 — Additional file 1:Tables S1–S7. Frequencies of metastasis and clinicopathologic characteristics in each histological subtype with or without nodal metastasis. [file 12876_2022_2163_MOESM1_ESM.pdf]

Supplementary Table 1 Frequencies of recurrence/metastasis in each histological subtype

	WMDA	PDA	MUA	SRCC
*Recurrence (%)	37/152 (24.3%)	34/98 (34.7%)	15/64 (23.4%)	3/9 (33.3%)
RR (95%CI)	Reference	1.425 (0.963-2.094)	0.963 (0.563-1.592)	1.369 (0.479-2.821)
***p* value		0.086	1.000	0.691

*In this study cohort, there were no cases of local recurrence throughout the histological subtypes. Therefore, recurrence means distant metastasis due to hematogenous/lymphatic metastasis or peritoneal dissemination. **Statistical analysis was performed by Fisher's exact test. *WMDA* well-to-moderately differentiated adenocarcinoma, *PDA* poorly differentiated adenocarcinoma, *MUA* mucinous adenocarcinoma, *SRCC* signet-ring cell carcinoma, *RR* relative risk, *CI* confidence interval.

Supplementary Table 2 Clinicopathologic characteristics of node-negative WMDA according to degree of venous invasion

Parameter	Degree of venous invasion			Total (n = 94)	*p value	
	v0 (n = 53)	v1 (n = 32)	v2 (n = 9)		v0 *vs.* v1	v0 *vs.* v2
Age						
≥ 65	40	19	6	65	0.148	0.683
< 65	13	13	3	29		
Gender						
Male	34	19	9	62	0.818	0.047
Female	19	13	0	32		
Family history of CRC						
Yes	3	1	0	4	1.000	1.000
No	50	31	9	90		
Location						
Left-sided	36	26	6	68	0.215	1.000
Right-sided	17	6	3	26		
Depth of invasion						
pTis/pT1/pT2	24	10	2	36	0.255	0.282
pT3/pT4	29	22	7	58		
Postoperarive chemotherapy						
Yes	7	6	3	16	0.543	0.151
No	46	26	6	78		
Postoperative radiation						
Yes	0	0	1	1	1.000	0.145
No	53	32	8	93		

*Statistical analysis was performed by Fisher's exact test. *WMDA* well-to-moderately differentiated adenocarcinoma, *CRC* colorectal cancer, *v0* no venous invasion, *v1* 1-3 invasions/glass slide, *v2* 4-6 invasions/glass slide, *v3* ≥7 invasions/glass slide. Filling type of venous invasion in macroscopically identifiable vein with a minor axis of ≥ 1 mm raised the grade of a v1 or v2 by 1.

Supplementary Table 3 Clinicopathologic characteristics of node-positive WMDA according to degree of venous invasion

Parameter	Degree of venous invasion				Total (n = 58)	*p value		
	v0 (n = 16)	v1 (n = 24)	v2 (n = 14)	v3 (n = 4)		v0 *vs.* v1	v0 *vs.* v2	v0 *vs.* v3
Age								
≥ 65	11	13	9	2	35	0.512	1.000	0.587
< 65	5	11	5	2	23			
Gender								
Male	12	11	8	2	33	0.104	0.442	0.549
Female	4	13	6	2	25			
Family history of CRC								
Yes	1	1	1	0	3	1.000	1.000	1.000
No	15	23	13	4	55			
Location								
Left-sided	13	14	9	3	39	0.177	0.417	1.000
Right-sided	3	10	5	1	19			
Depth of invasion								
pTis/pT1/pT2	1	3	0	0	4	0.638	1.000	1.000
pT3/pT4	15	21	14	4	54			
Postoperarive chemotherapy								
Yes	9	13	11	3	36	1.000	0.260	0.619
No	7	11	3	1	22			
Postoperative radiation								
Yes	0	1	2	1	4	1.000	0.209	0.200
No	16	23	12	3	54			

*Statistical analysis was performed by Fisher's exact test. *WMDA* well-to-moderately differentiated adenocarcinoma, *CRC* colorectal cancer, *v0* no venous invasion, *v1* 1-3 invasions/glass slide, *v2* 4-6 invasions/glass slide, *v3* ≥7 invasions/glass slide. Filling type of venous invasion in macroscopically identifiable vein with a minor axis of ≥ 1 mm raised the grade of a v1 or v2 by 1.

Supplementary Table 4 Clinicopathologic characteristics of node-negative PDA according to degree of venous invasion

Parameter	Degree of venous invasion			Total (n = 31)	*p value	
	v0 (n = 11)	v1 (n = 15)	v2+v3 (n = 5)		v0 *vs.* v1	v0 *vs.* v2+v3
Age						
≥ 65	9	10	1	20	0.658	0.036
< 65	2	5	4	11		
Gender						
Male	3	9	3	15	0.130	0.299
Female	8	6	2	16		
Family history of CRC						
Yes	0	0	0	0	1.000	1.000
No	11	15	5	31		
Location						
Left-sided	3	4	5	12	1.000	0.026
Right-sided	8	11	0	19		
Depth of invasion						
pTis/pT1/pT2	0	2	2	4	0.492	0.083
pT3/pT4	11	13	3	27		
Postoperative chemotherapy						
Yes	2	4	3	9	1.000	0.251
No	8	11	2	21		
Unknown	1	0	0	1		
Postoperative radiation						
Yes	1	0	0	1	0.400	1.000
No	9	15	5	29		
Unknown	1	0	0	1		

*Statistical analysis was performed by Fisher's exact test. *PDA* poorly differentiated adenocarcinoma, *CRC* colorectal cancer, *v0* no venous invasion, *v1* 1-3 invasions/glass slide, *v2* 4-6 invasions/glass slide, *v3* ≥7 invasions/glass slide. Filling type of venous invasion in macroscopically identifiable vein with a minor axis of ≥ 1 mm raised the grade of a v1 or v2 by 1.

Supplementary Table 5 Clinicopathologic characteristics of node-positive PDA according to degree of venous invasion

Parameter	Degree of venous invasion				Total (n = 67)	*p value		
	v0 (n = 7)	v1 (n = 37)	v2 (n = 14)	v3 (n = 9)		v0 *vs.* v1	v0 *vs.* v2	v0 *vs.* v3
Age								
≥ 65	3	27	9	4	43	0.184	0.397	1.000
< 65	4	10	5	5	24			
Gender								
Male	3	18	8	3	32	1.000	0.659	1.000
Female	4	19	6	6	35			
Family history of CRC								
Yes	0	0	0	1	1	1.000	1.000	1.000
No	7	37	13	8	65			
Unknown	0	0	1	0	1			
Location								
Left-sided	4	13	7	4	28	0.402	1.000	1.000
Right-sided	3	24	7	5	39			
Depth of invasion								
pTis/pT1/pT2	1	0	0	0	1	0.159	0.333	0.438
pT3/pT4	6	37	14	9	66			
Postoperarive chemotherapy								
Yes	4	21	7	7	39	1.000	0.642	1.000
No	2	16	7	2	27			
Unknown	1	0	0	0	1			
Postoperative radiation								
Yes	0	1	0	0	1	1.000	1.000	1.000
No	6	36	14	9	65			
Unknown	1	0	0	0	1			

*Statistical analysis was performed by Fisher's exact test. *PDA* poorly differentiated adenocarcinoma, *CRC* colorectal cancer, *v0* no venous invasion, *v1* 1-3 invasions/glass slide, *v2* 4-6 invasions/glass slide, *v3* ≥7 invasions/glass slide. Filling type of venous invasion in macroscopically identifiable vein with a minor axis of ≥ 1 mm raised the grade of a v1 or v2 by 1.

Supplementary Table 6 Clinicopathologic characteristics of node-negative MUA according to degree of venous invasion

Parameter	Degree of venous invasion		Total (n = 37)	**p* value
	v0 (n = 24)	v1 (n = 13)		v0 *vs.* v1
Age				
≥ 65	17	9	26	1.000
< 65	7	4	11	
Gender				
Male	20	8	28	0.229
Female	4	5	9	
Family history of CRC				
Yes	2	1	3	1.000
No	21	12	33	
Unknown	1	0	1	
Location				
Left-sided	13	5	18	0.495
Right-sided	11	8	19	
Depth of invasion				
pTis/pT1/pT2	4	0	4	0.276
pT3/pT4	20	13	33	
Postoperative chemotherapy				
Yes	4	4	8	0.433
No	18	9	27	
Unknown	2	0	2	
Postoperative radiation				
Yes	3	0	3	0.279
No	19	13	32	
Unknown	2	0	2	

*Statistical analysis was performed by Fisher's exact test. *MUA* mucinous adenocarcinoma, *CRC* colorectal cancer, *v0* no venous invasion, *v1* 1-3 invasions/glass slide, *v2* 4-6 invasions/glass slide, *v3* ≥7 invasions/glass slide. Filling type of venous invasion in macroscopically identifiable vein with a minor axis of ≥ 1 mm raised the grade of a v1 or v2 by 1.

Supplementary Table 7 Clinicopathologic characteristics of node-positive MUA according to degree of venous invasion

Parameter	Degree of venous invasion			Total (n = 26)	*p value	
	v0 (n = 9)	v1 (n = 13)	v2 (n = 4)		v0 *vs.* v1	v0 *vs.* v2
Age						
≥ 65	6	9	3	18	1.000	1.000
< 65	3	4	1	8		
Gender						
Male	5	8	1	14	1.000	0.559
Female	4	5	3	12		
Family history of CRC						
Yes	1	0	0	1	0.409	1.000
No	8	13	4	25		
Location						
Left-sided	4	9	1	14	0.384	1.000
Right-sided	5	4	3	12		
Depth of invasion						
pTis/pT1/pT2	1	0	1	2	0.409	1.000
pT3/pT4	8	13	3	24		
Postoperarive chemotherapy						
Yes	5	7	2	14	1.000	1.000
No	4	5	2	11		
Unknown	0	1	0	1		
Postoperative radiation						
Yes	0	0	0	0	1.000	1.000
No	9	12	4	25		
Unknown	0	1	0	1		

*Statistical analysis was performed by Fisher's exact test. *MUA* mucinous adenocarcinoma, *CRC* colorectal cancer, *v0* no venous invasion, *v1* 1-3 invasions/glass slide, *v2* 4-6 invasions/glass slide, *v3* ≥7 invasions/glass slide. Filling type of venous invasion in macroscopically identifiable vein with a minor axis of ≥ 1 mm raised the grade of a v1 or v2 by 1.
